# The influence of Gleason score ≤ 6 histology on the outcome of high-risk localized prostate cancer after modern radiotherapy

**DOI:** 10.1038/s41598-024-55457-z

**Published:** 2024-04-05

**Authors:** Hideya Yamazaki, Gen Suzuki, Koji Masui, Norihiro Aibe, Takuya Kimoto, Kei Yamada, Koji Okihara, Fumiya Hongo, Masayoshi Okumi, Takumi Shiraishi, Atsuko Fujihara, Ken Yoshida, Satoaki Nakamura, Takashi Kato, Yasutoshi Hashimoto, Haruumi Okabe

**Affiliations:** 1 Department of Radiology, Graduate School of Medical Science, Kyoto Prefectural University of Medicine, 465 Kajiicho Kawaramachi Hirokoji, Kamigyo-ku, Kyoto, Kyoto 602-8566 Japan; 2 Department of Urology, Graduate School of Medical Science, Kyoto Prefectural University of Medicine, 465 Kajiicho Kawaramachi Hirokoji, Kamigyo-ku, Kyoto, 602-8566 Japan; 3 Department of Department of Radiology, Kansai Medical University, Hirakata, 573-1010 Japan; 4 Department of Radiology, Ujitakeda Hospital, Uji-City, Kyoto 611-0021 Japan

**Keywords:** Prostate cancer, High-dose-rate brachytherapy, Low-dose-rate brachytherapy, Intensity modulated radiotherapy, Stereotactic body radiotherapy, Gleason score, Oncology, Urology

## Abstract

We aimed to retrospectively review outcomes in patients with high-risk prostate cancer and a Gleason score ≤ 6 following modern radiotherapy. We analyzed the outcomes of 1374 patients who had undergone modern radiotherapy, comprising a high-risk low grade [HRLG] group (Gleason score ≤ 6; n = 94) and a high-risk high grade [HRHG] group (Gleason score ≥ 7, n = 1125). We included 955 patients who received brachytherapy with or without external beam radio-therapy (EBRT) and 264 who received modern EBRT (intensity-modulated radiotherapy [IMRT] or stereotactic body radiotherapy [SBRT]). At a median follow-up of 60 (2–177) months, actuarial 5-year biochemical failure-free survival rates were 97.8 and 91.8% (p = 0.017), respectively. The frequency of clinical failure in the HRLG group was less than that in the HRHG group (0% vs 5.4%, p = 0.012). The HRLG group had a better 5-year distant metastasis-free survival than the HRHG group (100% vs 96.0%, p = 0.035). As the HRLG group exhibited no clinical failure and better outcomes than the HRHG group, the HRLG group might potentially be classified as a lower-risk group.

## Introduction

Prostate cancer is the most diagnosed malignancy among men in Western countries^1^. In 2021, approximately 248,530 new cases of prostate cancer were diagnosed in the United States, accounting for 10.7% of cancer-related deaths^1^. Prostate-specific antigen (PSA) screening systems have facilitated its early detection. Low-grade (Gleason score ≤ 6) prostate cancer, the most common subgroup^1–4^, may not threaten life expectancy in general, while overtreatment, which can induce unnecessary side-effects, might affect quality of life and substantially increase healthcare costs^4^. To reduce the risk of overtreatment, active surveillance (AS) has been introduced as a standard of care for patients with low-risk prostate cancer and a life expectancy of ≥ 10 years^1^.

Some studies have suggested that prostate cancers with a Gleason score of ≤ 6 should be considered as non-cancerous lesions that need not be treated^4–6^, while other studies have not supported this suggestion^7–9^ given the pathological, surgical, and radiotherapeutic aspects involved in diagnosing and treating such patients^10–13^. To our knowledge, few studies have focused on outcome data in relation to high-risk patients with a Gleason score ≤ 6 following radiotherapy using advanced technology, namely, intensity modulated radiotherapy (IMRT), stereotactic body radiotherapy (SBRT), and/or brachytherapy (BT) using higher prescribed doses sufficient to meet modern radiotherapy criteria^1^.

Dose escalation has been shown to improve biochemical control in patients with localized prostate cancer^14–17^, and the National Comprehensive Cancer Network guidelines recommend doses > 70 Gy in conventional fractions^1^. Therefore, we examined the role of a Gleason score ≤ 6 in patients with high-risk prostate cancer treated with modern radiotherapy (IMRT) using a high dose and/or advanced technology (SBRT or BT).

We used freely available public data to analyze a large cohort on high-dose rate BT (HDR-BT) and external beam radiotherapy (EBRT)^18,19^, SBRT^18,20^, and IMRT. We combined data of low-dose rate brachytherapy (LDR-BT) with or without EBRT^21^ and IMRT^22^ performed in our institutions. We aimed to compare modern radiotherapy outcomes between patients with high-risk low grade (HRLG, Gleason score ≤ 6) prostate cancer and those with high-risk higher grades (HRHG, Gleason score ≥ 7) prostate cancer.

## Materials and methods

### Patients

We retrospectively examined the data of patients treated with radiotherapy, including BT with or without EBRT and modern EBRT (IMRT and SBRT). Data of 916 patients treated with HDR-BT, 67 treated with SBRT, and 132 treated with IMRT were obtained from open databases for public use^18–20^. We also obtained data of 39 patients who had been treated with LDR-BT at Kyoto Prefectural Medical School^23^ and 65 patients who had been treated with IMRT at Uji Takeda Hospital^22^. We included patients with histology-confirmed prostate-related adenocarcinoma with clinical T1–T4N0M0 disease, with available and accessible data on the Gleason score, pretreatment PSA (initial PSA [iPSA]) level, and T classification according to the NCCN risk classifications^1^. We excluded patients with node positivity, distant metastasis, a prescribed dose of ≤ 74 Gy in EQD2, and patients with missing data.

Biochemical failure was defined in accordance with the Phoenix ASTRO consensus (Nadir + 2 ng/ml). Clinical recurrence included local recurrence, pelvic lymph node recurrence, and distant metastasis. Patients with imaging evidence confirming clinically or pathologically diagnosed metastatic lesions were classified as exhibiting clinical recurrence. Prostate cancer-specific mortality (PCSM) was defined based on documentation of prostate cancer as a primary cause of death on the death certificate or clinical documentation. Outcomes of interest included biochemical disease-free survival rate (bDFS), distant metastasis-free survival (DMSF), prostate cancer specific mortality (PCSM), and overall survival (OS), which were defined in intervals from the start of radiotherapy to PSA failure, occurrence of distant metastasis, PCSM, and all causes of death, respectively. Informed consent was obtained by providing an opt-out form on the website, and patients who refused to participate were excluded. Furthermore, all patients included in our analysis from Kyoto Prefectural Medical School and Uji Takeda Hospital provided written informed consent. This study was conducted in accordance with the Declaration of Helsinki principles and was approved by the institutional review board of the Kyoto Prefectural University of Medicine (ERB-C-1403).

Using data available in the public domain, we analyzed a large cohort of patients who received HDR-BT with EBRT^18,19^, SBRT^18,20^, and partial IMRT. We combined the data from the LDR-BT with or without EBRT^21^ and IMRT^22^ interventions performed at our institutions.

### Treatment

We included 352 patients who had been treated with IMRT and 67 treated with SBRT using ˃ 70 Gy in equivalent doses of 2 Gy fractions (EQD2Gy) (n × d ([α/β] + d)/([α/β] + 2); n = number of treatment fractions; d = dose per fraction in Gy, α/β = 1.5 Gy). Details concerning treatment schedules are shown in Supplementary Table 1. IMRT data were obtained from a freely accessible dataset (n = 205)^18^, and 147 image-guided IMRTs using helical tomotherapy were performed at the Department of Radiology, Uji Takeda Hospital^22^. Image-guided IMRT techniques using helical tomotherapy and SBRT have been described elsewhere^20,22^. Briefly, during the initial period (from June 2007 to 2009), tomotherapy was performed at a prescribed dose of 74.8 Gy/34 fractions (2.2 Gy/fraction) for high- and intermediate-risk groups and 72.6 Gy for the low-risk group at 95% of the planning target volume receiving at least the prescribed dose. From 2009 to 2013, we reduced the dose to 74 Gy/37 fractions (2 Gy/fraction)^24^. IMRT treatment schedules were as follows: 74 Gy/37 fractions (n = 82), 72 Gy/36 fractions (n = 70), 74.8 Gy/34 fractions (n = 65), 70 Gy/28 fractions (n = 45), 78 Gy/39 fractions (n = 50), 80 Gy/40 fractions (n = 34), 72 Gy/35 fractions (n = 70), 67.5 Gy/27 fractions (n = 1), 62 Gy/20 fractions (n = 2), 65 Gy/26 fractions (n = 2), and 76 Gy/40 fractions (n = 1). The median prescribed IMRT dose was 72 Gy (62–80 Gy) in 36 fractions (range 20–40 fractions).

SBRT treatment schedules were as follows: 36 Gy/4 fractions (n = 37), 36.25 Gy/5 fractions (n = 8), and 35 Gy/5 fractions (n = 22). The median prescribed SBRT dose was 36 Gy (range 32–36 Gy) in 4 fractions (range 4–5 fractions) (Supplementary Table 1).

Considering BT, 39 patients underwent LDR-BT with or without EBRT and 916 underwent HDR-BT with EBRT. For the HDR plus EBRT group, we used multi-institution data from an open data source^18^, with treatment details described elsewhere^19,20^. HDR-BT treatment schedules were as follows: HDR-BT 31.5 Gy/5 fractions plus EBRT 30 Gy/10 fractions (n = 516), HDR-BT 18 Gy/2 fractions (n = 208) plus EBRT 39 Gy/13 fractions or 51 Gy/17 fractions or 48 Gy/16 fractions, HDR-BT 11 Gy/1 fraction plus EBRT 51 Gy/17 fractions (n = 103), HDR-BT 21 Gy/2 fractions or HDR-BT 21 Gy/3 fractions (n = 28) plus EBRT 45 Gy/15 fractions, or EBRT 42 Gy/14 fractions or EBRT 51 Gy/17 fractions, HDR-BT 20 Gy/2 fractions (n = 20) plus EBRT 46 Gy/23 fractions or EBRT 30 Gy/15 fractions, HDR-BT 25 Gy/5 fractions plus EBRT 51 Gy/17 fractions (n = 5), and HDR-BT 10.5 Gy/1 fraction plus EBRT 51 Gy/17 fractions (n = 1) (Supplementary Table 1). The median HDR-BT dose was 31.5 Gy (range 10.5–31.5 Gy), and the median fraction size was 6.3 Gy (range 5–11 Gy) using additional EBRT in various fractions (median dose, 31.5 Gy; median fraction size, 3 Gy; range 2–3 Gy). Considering LDR-BT with or without EBRT, we used a prescribed dose of 145 Gy (Gleason score ≤ 6) or 110 Gy (Gleason score ≥ 7, with 40 Gy/20 fractions EBRT)^21^.

### Statistical analyses

StatView 5.0 and EZR statistical software were used for all statistical analyses^23^. For percentage comparisons, Fisher’s exact test was used, and the Mann–Whitney U-test was used to compare means or medians. To analyze survival data, the Kaplan–Meier method was used for DFS, DMSF, PCSM, and OS. A log-rank test was used for comparisons. Cause-specific manner (death due to another cause of cancer was assigned as a censor) was applied to bDFS, DMSF, and PCSM. Cox’s proportional hazard model was used for uni- and multivariate analyses for bDFS. For the univariate analysis of bDFS, the following factors were evaluated: BT vs. EBRT, androgen deprivation therapy ([ADT], yes vs. no), age (≤ 74 years vs. ≥ 75 years), T category (T1–2a vs. T2b-4), iPSA (≤ 10 ng/ml vs. > 10 ng/ml), and Gleason score (≤ 6 or ≥ 7). For multivariate analysis of bDFS, the following factors were evaluated: BT vs. EBRT, T category (T1–2a vs. T2b-4) and Gleason score (≤ 6 or ≥ 7), which were identified as statistically significant factors in univariate analysis. A p-value < 0.05 was considered statistically significant.

Given that included patients did not undergo randomization, unbalanced baseline characteristics existed, which could have led to a selection bias. Therefore, we used the propensity score, which was defined as the probability of being allocated to the HGLG or HGHG groups, considering patient characteristics. The logistic regression model was used to calculate propensity scores, considering the baseline covariates shown in Table 2 (age, BT ± EBRT or EBRT, T classification, iPSA, and hormonal therapy). After initial analysis of the entire cohort, we created a propensity score-matched pair cohort to minimize bias related to allocation to the HGLG or HGHG groups. Five selected factors for the whole cohort were used to establish a 1:10 matched cohort assigned to the HGLG or HGHG groups, given that the HRHG group comprised nearly 10 times more patients than the HRLG group in the original cohort. Next, to validate our findings, we generated another propensity score-matched paired cohort with a 1:10 HRLG ratio for patients with HRHG. Finally, a sensitivity analysis was performed, generating three additional matched pairs with a 1:10 patient ratio, whose BT ± EBRT or EBRT, age, and hormonal therapy factors had been eliminated from the original 5 adjustment factors, given that these three factors did not show any statistically significant differences between the HRLG and HRHG groups in the original cohort (Table 1).

**Table 1:** Background patient characteristics between patients with Gleason score ≤ 6 (HRLGH) and Gleason score ≥ 7 (HRHG).

Variables	Strata	HRLG	HRHG	*p*-value
		No. (%) or median [range]	No. (%) or median [range]	
		(n = 94)	(n = 1125)	
Age		71 [54, 86]	70.3 [48, 86]	0.878
Gleason score	5	8 (8.5)	0 (0.0)	** < 0.001**
	6	86 (91.5)	0 (0.0)	
	7	0 (0.0)	426 (37.9)	
	8 ≤	0 (0.0)	699 (62.1)	
ADT	No	10 (10.6)	68 (6.0)	0.119
	Yes	84 (89.4)	1057 (94.0)	
Modality (%)	HDR + EBRT	61 (64.9)	855 (76.0)	** < 0.001**
	IMRT	21 (22.3)	176 (15.6)	
	LDR ± EBRT	11 (11.7)	28 (2.5)	
	SBRT	1 (1.1)	66 (5.9)	
iPSA	ng/ml	20.26 [4.55, 107]	17.40 [2.68, 1454]	0.697
T category	1	14 (14.9)	149 (13.2)	0.828
	2	31 (33.0)	372 (33.1)	
	3	48 (51.1)	595 (52.9)	
	4	1 (1.1)	9 (0.8)	

iPSA = initial PSA value, % of HRLG in BT ± EBRT and EBRT = 7.5% and 8.3% (p = 0.696).

Significant values are in bold.

### Institutional review board statement

The study was conducted according to the guidelines of the Declaration of Helsinki and approved by the Institutional Review Board of Kyoto Prefectural University of Medicine: ERB-C-1330-3.

### Informed consent statement

Informed consent was obtained from all subjects involved in the study.

## Results

### Patients and treatment characteristics

The patient median age was 70 (range 48–86) years, and the median follow-up period for the entire cohort was 60 months (range 2–177 months), with a 1-year minimum for surviving patients or until death. Table 1 compares the clinicopathological background characteristics of the two groups (HRLG and HRHG groups). The HRHG group included patients with advanced disease who required more hormonal therapy than patients in the HRLG group.

### Assessment of biochemical disease-free survival (bDFS)

**Figure 1:**
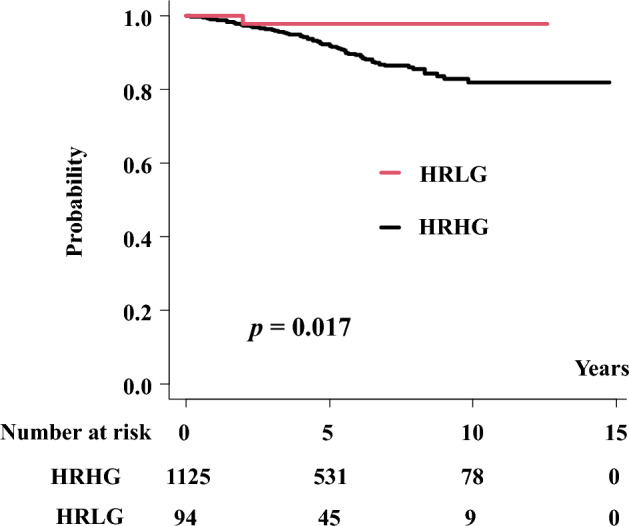
Biochemical disease-free survival (bDFS).

Overall, 2 (2.1%) patients in HRLG developed biochemical failure when compared with 101 (9.0%) patients in HRHG (p = 0.019). The actuarial 5-year bDFS values were 97.8% (95% confidential interval [CI] 91.6–99.5%) and 91.8% (89.7–93.5%, p = 0.017, Fig. 1) in HRLG and HRHG, respectively. We noted a significant difference in the biochemical control rate between HRLG and HRHG. As shown in Table 2, we identified BT vs. EBRT, T category (T1–2a vs. T2b-4), and Gleason score (HRLG vs. HRHG) as statistically significant factors for bDFS in uni- and multivariate Cox regression analyses. A Gleason score ≤ 6 showed the lowest hazard ratio 0.20 among these influential factors (Table 2). No patient with a Gleason score of 5 (categorized as non-cancerous in 2005^4^) showed PSA failure, clinical failure, or distant metastasis.

**Table 2:** Uni- and multivariate analysis of biochemical control rate.

Variables	Univariate		Multivariate	
	Hazard ratio (95% CI)	*p*-value	Hazard ratio (95% CI)	*p*-value
BT vs EBRT	1.49 (0.91–2.44)	0.105	1.55 (0.93–2.58)	0.086
ADT: yes vs no	0.95 (0.42–2.18)	0.919	0.73 (0.30–1.73)	0.476
Age ≤ 74 vs 75 ≤	1.07 (0.66–1.72)	0.768	1.05 (0.65–1.70)	0.835
T1–2a vs T2b-4	0.50 (0.29–0.87)	**0.014**	0.50 (0.29–0.88)	**0.017**
iPSA ≤ 10 ng/ml vs 10 < ng/ml	0.57 (0.33–0.98)	0.043	0.59 (0.34–1.02)	0.060
HRLG vs HRHG	0.21 (0.05–0.87)	**0.031**	0.20 (0.05–0.83)	**0.027**

Significant values are in bold.

### Comparison of HRLG and HRHG in relation to clinical outcomes

A follow-up of this study cohort identified 61 (5.4%) documented clinical failures in the HRHG group and 0 in the HRLG group (p = 0.012).

**Figure 2:**
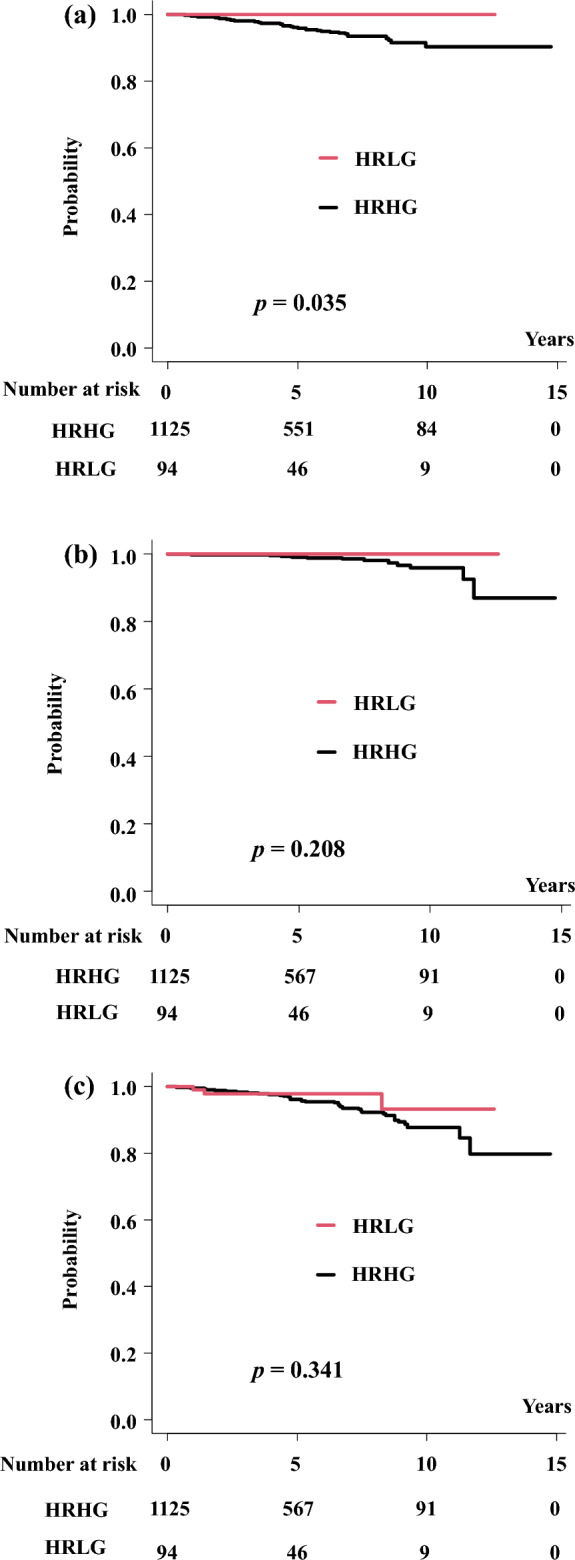
(**a**) Distant metastasis free survival (DMSF). (**b**) Prostate cancer-specific survival rate (PCSM). (**c**) Overall survival rate (OS).

Fifty patients had distant metastases (HRLG group, n = 0; HRHG group, n = 50, p = 0.083), and the 5-year [10-year] DMSF rates were 100% [100%] in the HRLG group and 96.0% [90.4%] (94.3–97.1% [86.2–93.4%]) (p = 0.035) in the HRHG group (Fig. 2a). There was a significant difference in the DMSF between the HRLG and HRHG groups. No clinical recurrence was observed in the HRLG group nor were there any prostate cancer-related deaths.

We identified 16 prostate cancer-related deaths in this cohort, all of which involved patients in the HRHG group. The 5-year [10-year] PCSM values were 100% [100%] and 98.9% [95.9%] in the HRLG and HRHG groups, respectively (95% CI 97.8–99.5% and 92.1–97.9%, respectively, p = 0.208, Fig. 2b). There was a statistically significant difference in the PCSM values between the HRLG and HRHG groups.

The overall 5-year [10-year] survival rates were 97.9% [93.2%] (95% CI 91.8–99.5% [74.7–98.3%]) and 96.0% [87.8%] (94.4–97.2% [83.1–91.2%], p = 0.341) in the HRLG and HRHG, respectively (Fig. 2c).

### Comparison of outcomes between HRHG and HRLG groups using the propensity score matched pair cohort

**Figure 3:**
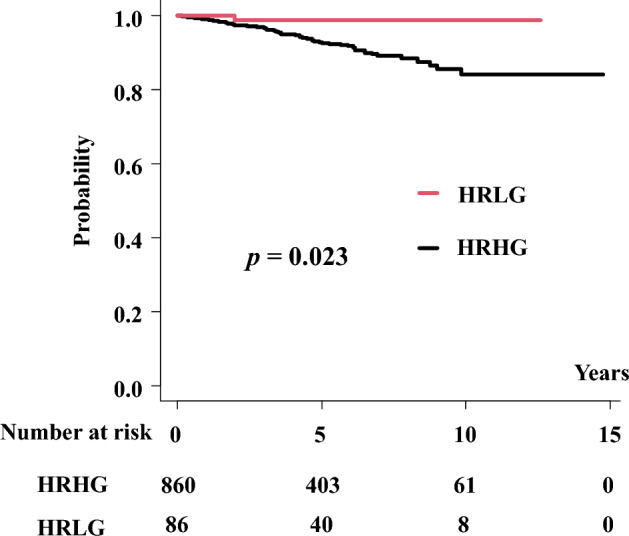
Biochemical disease-free survival (bDFS) for propensity-score matched-pair cohort.

By matching the propensity scores, we obtained a well-matched set of 86:860 patient pairs in the HGLG and HGHG groups from the original cohort. Supplementary Table 2 presents a comparison of patient background characteristics. The HGLG group had a superior 5-year bDFS rate to that of the HRHG group (98.8% vs. 92.5%, respectively, p = 0.023; Fig. 3). The 5-year DMSF rates were 100 and 96.5% for the HRLG and HRHG groups, respectively (p = 0.072; Supplementary Fig. 1a). The 5-year PCSM rates were 100 and 99.0% for the HRLG and HRHG group, respectively (p = 0.283; Supplementary Fig. 1b). The 5-year OS rates were 97.7 and 96.6% for the HRLG and HRHG groups, respectively (p = 0.601; Supplementary Fig. 1c).

Based on the results of the sensitivity analysis, the HRLG group exhibited a significantly better bDFS than the HRHG group upon eliminating BT vs. EBRT (HR 0.212, 95% CI 0.052–0.862; p = 0.03), along with a marginal significance detected on eliminating hormonal factor (HR 0.255, 95% CI 0.062–1.04; p = 0.068) or the age factor (HR 0.145, 95% CI 0.020–1.04; p = 0.055).

## Discussion

We assessed data from 1219 males who had undergone modern radiotherapy and found that those in the HRLG group had better outcomes than those in the HRHG group. The indolent nature of HRLG cancer lesions was confirmed in terms of their outcomes, in that no clinical failures nor prostate cancer-related deaths occurred in patients in the HRLG group. The outcomes for patients in the HRLG group were equivalent to those of an intermediate-risk group^22^, even in the presence of high-risk factors, such as T3–4 or iPSA ˃20 ng/ml. Accordingly, we speculate the potential for downstaging the patients with HRLG prostate cancer to a lower-risk group. At least, no further intensive treatment (whole pelvic radiotherapy, boost radiotherapy using BT, new drug etc.) would be required for HRLG. Our findings may be beneficial for counseling individual patients with HRLG lesions with respect to their treatment choices and prognoses.

More than 50 years ago, Donald Gleason introduced a grading system for prostate cancer, and the Gleason scoring system has remained an essential tool to predict disease outcomes and to facilitate clinical decision-making^24^. Several studies have confirmed the importance of this grading system concerning its predictive value in relation to prostate cancer mortality (Gleason score ˃8–10), as well as showing the indolent nature of prostate cancer lesions with a Gleason score ≤ 6^1–10^. While a Gleason score ≤ 6 includes neoplasia from a histological perspective, it is typically indicative of an indolent non-neoplastic precursor lesion. Reclassifying this type of lesion as non-cancerous re-mains controversial; however, such reclassification could help to minimize overtreatment, and reduce treatment-related side-effects, patient anxiety, and the financial burden to healthcare systems. Kweldam et al. reported no disease-specific death or metastasis in patients with a Gleason score ≤ 6 following radical prostatectomy^11^. Eggener et al. observed that only 3 of 9557 patients with localized prostate cancer and a Gleason score ≤ 6 had died during a 15-year follow-up period^12^. Finally, several patients with Gleason scores ≤ 6 reportedly did not require immediate intervention and were often eligible for AS^1,2^.

Underestimation of high-grade cancer cases after prostate needle biopsy remains an important issue. As prostate cancer is a heterogeneous disease, it is estimated that 30–50% of patients with low-risk prostate cancer and 13% with very low-risk prostate cancer exhibit occult high-grade disease on surgical pathology after radical prostatectomy^25^. Epstein et al. reported that 36% of cases (1841/5071) were upgraded from a needle biopsy derived Gleason score of 6 to a higher grade following surgical pathological ex-amination^25,26^. Leeman et al. demonstrated the role of advanced age in upgrading and upstaging in patients with prostate cancer and with a Gleason score of 6 after radical prostatectomy. Of 3571 patients analyzed, 115 (3.22%), 245 (6.86%), and 254 (7.11%) were upgraded, upstaged, or had positive surgical margins, respectively, with advanced age at diagnosis being associated with an increased risk of upgrading the disease to a Gleason score ≥ 7 after prostatectomy T3/T4 and positive surgical margins (adjusted odds ratios, 1.05, 1.02, and 1.02, respectively). Similarly, advanced age was associated with an increasing number of men with disease upgraded to a Gleason score ≥ 7 or upstaged to pT3/4- or pT2-positive surgical margins among those with 33% positive biopsy scores (but not < 33%), following staging using multiparametric magnetic resonance imaging (MRI). Therefore, multiparametric MRI should be considered in healthy older men with a Gleason score ≤ 6 and ≥ 33% positive biopsy cores. Almost all Japanese institutions perform systematic random biopsies (≥ 12 samples). Recently, MRI/US fusion-guided biopsy was introduced and used simultaneously with systematic random biopsy in our institution. Yamada et al. reported that MRI-targeted biopsy is superior to standard systematic biopsy for the detection of clinically notable cancers. However, given that certain prominent cancers can be detected by standard systematic biopsy only, a combination of standard systematic biopsy with MRI-targeted biopsy would avoid the underdiagnosis of clinically relevant cancers^27^.

Furthermore, underestimation can be a more significant factor in radiotherapy than in surgery as pathological specimens are not obtained during radiotherapy, especially with radiotherapy usually applied in more advanced disease than surgery. Therefore, several investigations have been undertaken to improve biopsy quality. Pepe et al. re-ported good accuracy for quantitative histological examinations in predicting non-localized prostate cancer using other biopsy parameters^28^. Ngnen et al. reviewed biopsy-based genomic classifier (GC: gene expression biomarker) in high-risk prostate cancer from prospective randomized trials and reported an independent association between the GC score with DMSF, PCSM, and OS^29^. Long term follow-up of randomized trials has shown that > 70% of men with high-risk prostate cancer after definitive radiation therapy and long-term ADT will never develop metastatic disease^29^. Thus, Ngnen et al. concluded that high-risk prostate cancer is a heterogeneous disease state, and using the GC score can improve risk stratification to facilitate personalized shared decision making^29^. Our data might also be useful to improve risk stratification in relation to high-risk prostate cancer using this simple method.

The probability of grade-up transformations, which involves progression in a Gleason score ≤ 6 to high-grade disease, should also be considered^30^. Molecular data suggest that genomic instability, which causes tumor progression, precedes the detection of histologically visible changes, and the potential risk of metastasis or mortality from prostate cancer with a Gleason score of 6 should be considered^30^. In a cohort of extensive AS studies, up to 33% of patients with Gleason scores of 6 required therapeutic intervention, primarily owing to upgrading^2,31^. Tosoian et al. examined a prospective AS cohort of 1,818 patients at a single institution using multiparametric MRI and ultra-sound fusion-targeted biopsy and found that the risk of cancer death or metastasis was < 1% over a long-term follow-up period^31^. Moreover, recent advanced technologies, such as prostate-specific membrane antigen positron emission tomography imaging, can alter AS conditions.

Conversely, low-risk patients with a Gleason score ≤ 6 may be good candidates for AS. Based on a Japanese national survey, approximately 90% of urologists proposed AS for low-risk disease^32^, and the number of AS cases has increased over the past decades in Japan. However, not all physicians (urologists) recommend AS, even for low-risk patients. In addition, some patients request treatment even if they have a Gleason score ≤ 6 because they fear disease progression, especially younger patients. Kato et al. conducted a national questionnaire survey of Japanese urologists (involving 922 urologists at Japanese Urological Association Teaching Base Hospitals) regarding AS for patients with low- and intermediate-risk prostate cancer^32^ and reported that 90.5%, 90%, 39.5%, 48.7%, 15%, and 22% urologists proposed AS for patients in low-risk/no comorbidity, low-risk/with comorbidity, intermediate-risk 3 + 4/no comorbidity, intermediate-risk 3 + 4/with comorbidity, intermediate-risk 4 + 3/no comorbidity, and intermediate-risk 4 + 3/with comorbidity groups, respectively. Therefore, approximately 40% of these urologists proposed AS for intermediate-risk cases.

This study had some limitations. First, it was a retrospective and multi-institutional study with substantial heterogeneity, therefore, further validation is required to confirm our results, including patients with no radiotherapy and/or a population from Western countries, preferably in a prospective fashion. Additionally, different biopsy methods were used, ranging from random to 12-core biopsy, as well as recent multiparametric MRI-fused biopsy methods at several institutions. Moreover, data are limited concerning the core number of positive cases in public databases. Additionally, there is a lack of consensus concerning grading among pathologists owing to the absence of a central pathological review system, which has resulted in different follow-up protocols and treatment modalities. Therefore, to obtain more accurate results, studies with a relatively longer follow-up period with a larger patient cohort are required. Despite these limitations, to our knowledge, this is the first and largest comprehensive study to analyze the usefulness of Gleason scores ≤ 6 compared with Gleason scores ≥ 7 in terms of modern radiotherapy outcomes in patient with high-risk prostate cancer.

## Conclusions

The HRLG group exhibited better outcomes than the HRHG group. Patients classified into a high-risk group with a Gleason score ≤ 6 have the potential to be classified into a lower-risk group.

### Supplementary Information

Supplementary Figure 1.

Supplementary Tables.

## Data Availability

The datasets used and/or analysed during the current study are available from the corresponding author on reasonable request.
