# Supplementary material for: The influence of Gleason score ≤ 6 histology on the outcome of high-risk localized prostate cancer after modern radiotherapy
Source: Sci Rep. 2024 Apr 5;14:8011. doi: 10.1038/s41598-024-55457-z (PMC10997615; doi:10.1038/s41598-024-55457-z)
Supplement: Supplementary file 1 — Supplementary Figure 1. [file 41598_2024_55457_MOESM1_ESM.pptx]

## Slide 1
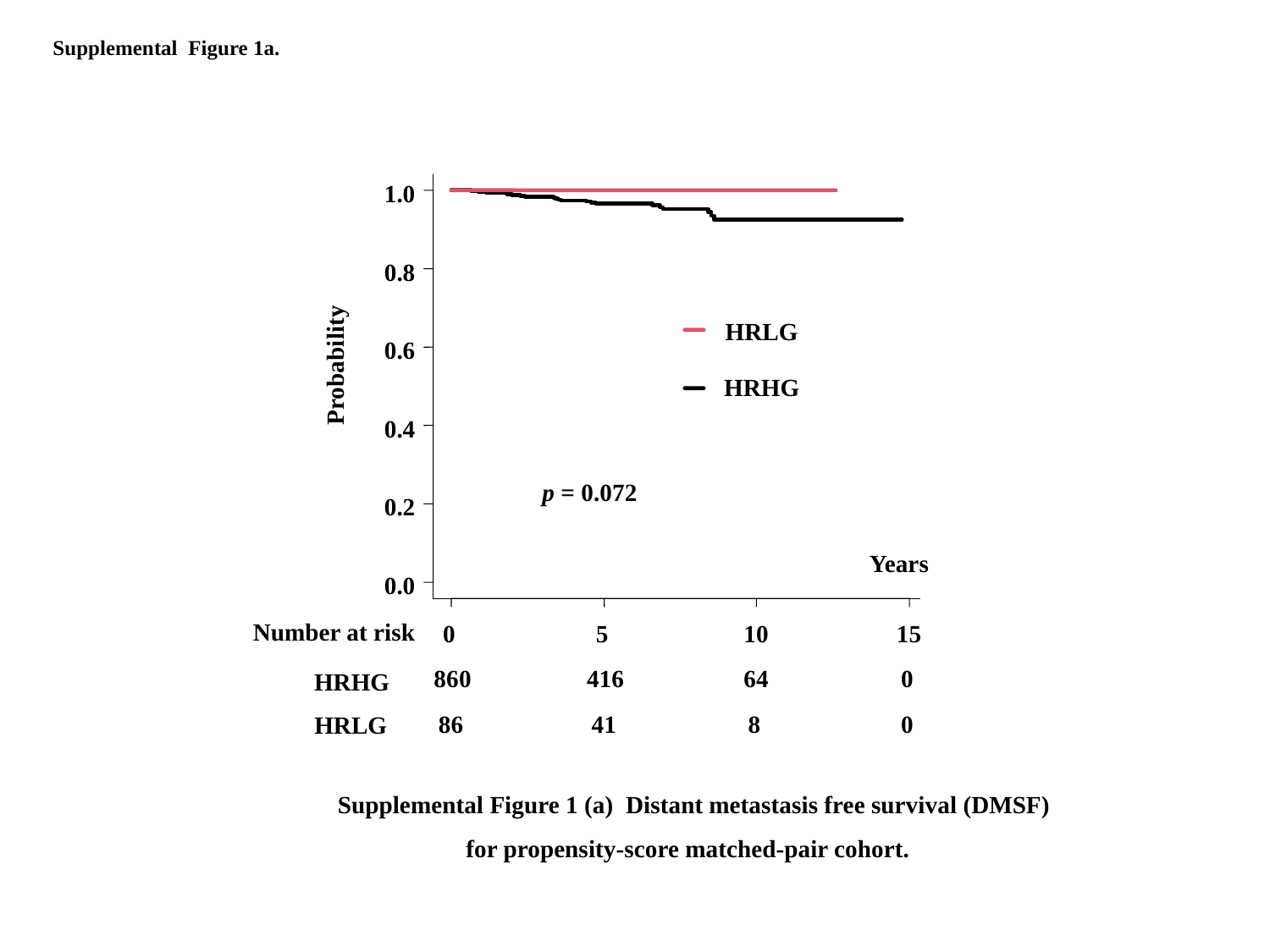

Supplemental Figure 1a.
1.0
0.8
HRLG
0.6
Probability
HRHG
0.4
p = 0.072
0.2
Years
0.0
Number at risk
0
5
10
15
HRHG
860
416
64
0
HRLG
86
41
8
0
Supplemental Figure 1 (a) Distant metastasis free survival (DMSF) for propensity-score matched-pair cohort.

## Slide 2
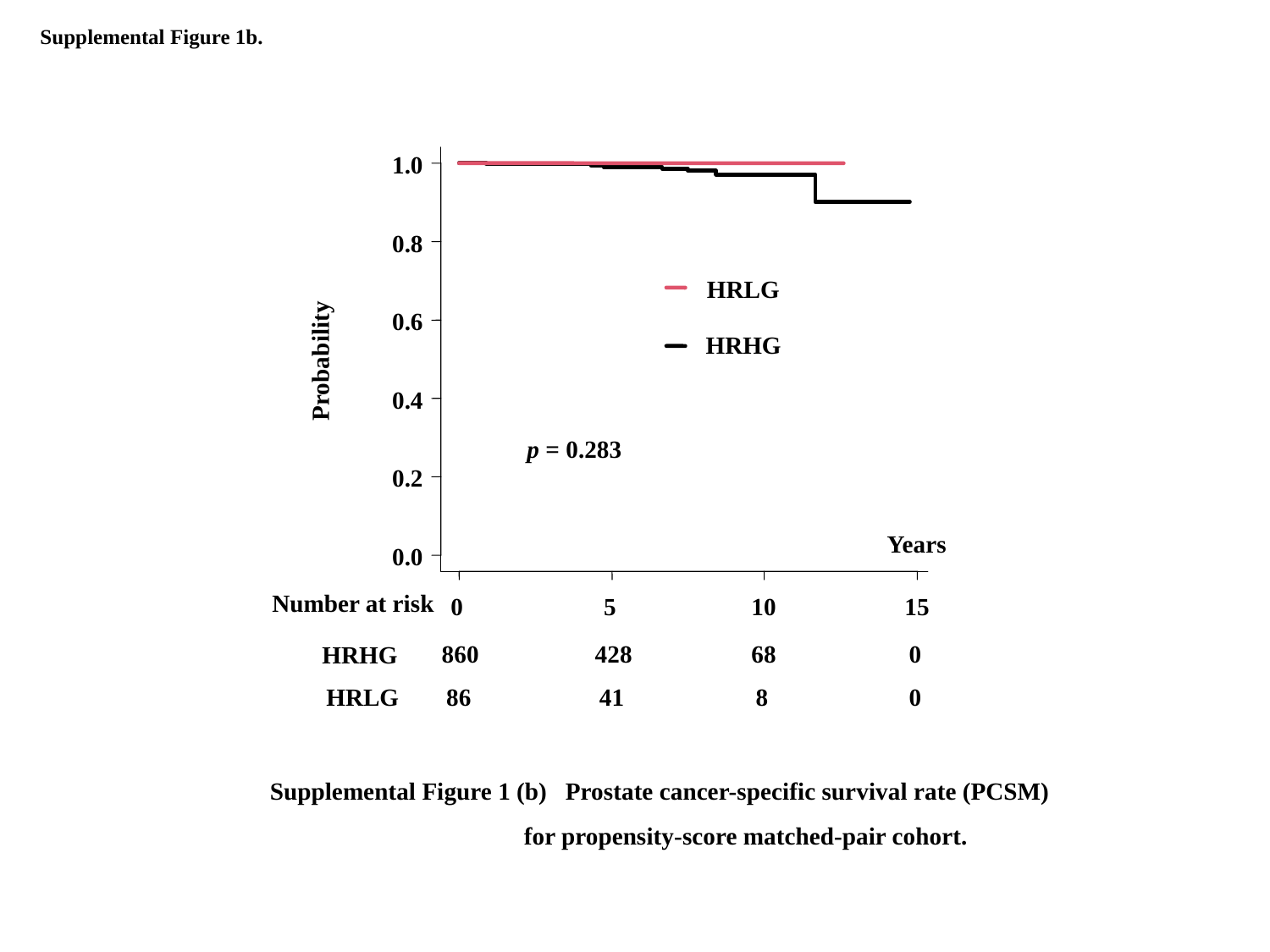

Supplemental Figure 1b.
1.0
0.8
HRLG
0.6
HRHG
Probability
0.4
p = 0.283
0.2
Years
0.0
Number at risk
0
5
10
15
HRHG
860
428
68
0
HRLG
86
41
8
0
Supplemental Figure 1 (b) Prostate cancer-specific survival rate (PCSM) 			for propensity-score matched-pair cohort.

## Slide 3
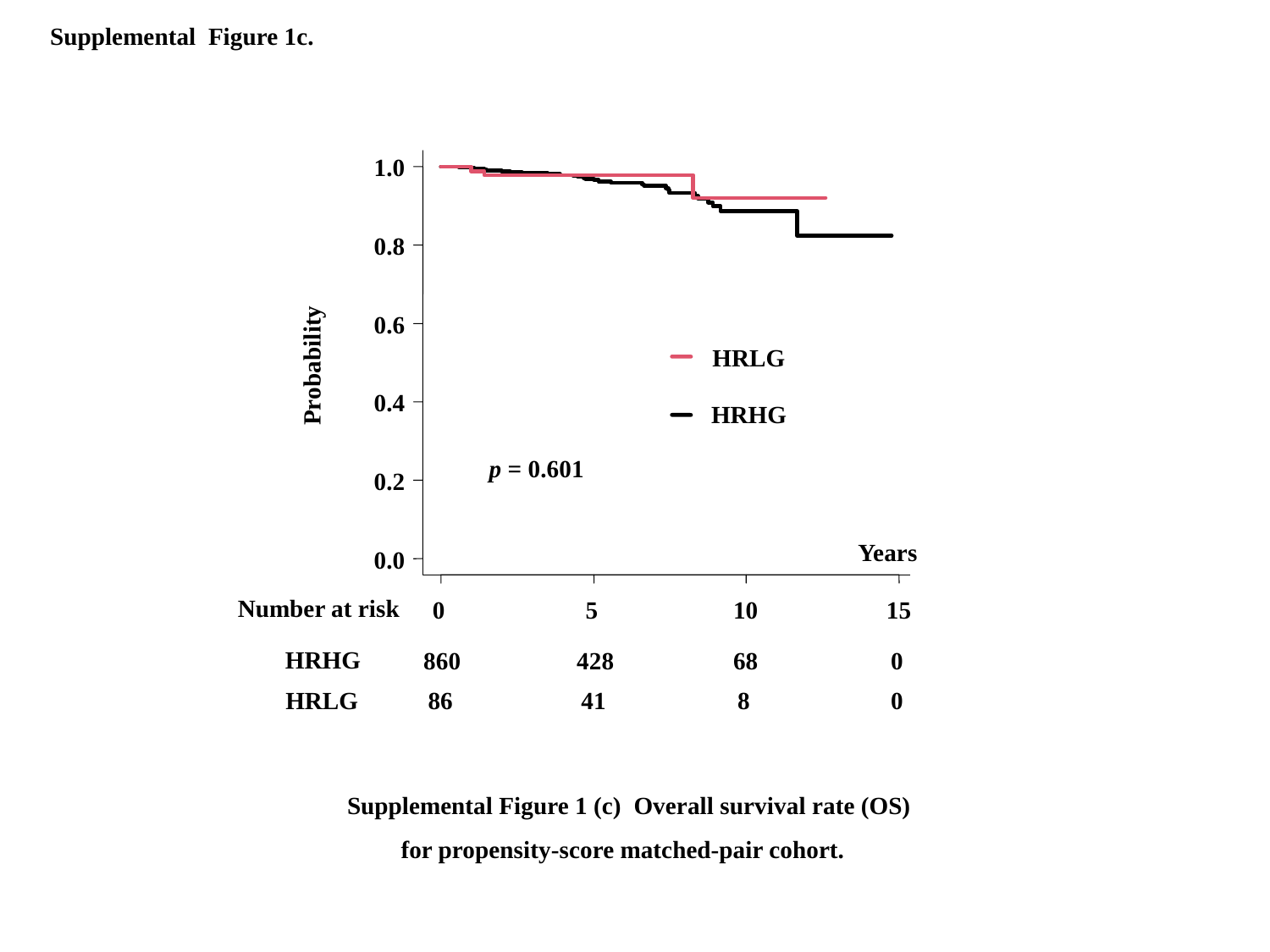

Supplemental Figure 1c.
1.0
0.8
0.6
HRLG
Probability
0.4
HRHG
p = 0.601
0.2
Years
0.0
Number at risk
0
5
10
15
HRHG
860
428
68
0
HRLG
86
41
8
0
Supplemental Figure 1 (c) Overall survival rate (OS) for propensity-score matched-pair cohort.
